# Cardiometabolic Risk Markers in Indian Children: Comparison with UK Indian and White European Children

**DOI:** 10.1371/journal.pone.0036236

**Published:** 2012-04-27

**Authors:** Claire M. Nightingale, Ghattu V. Krishnaveni, Alicja R. Rudnicka, Christopher G. Owen, Sargoor R. Veena, Jacqueline C. Hill, Derek G. Cook, Caroline H. D. Fall, Peter H. Whincup

**Affiliations:** 1 Division of Population Health Sciences and Education, St George's University of London, London, United Kingdom; 2 Epidemiology Research Unit, CSI Holdsworth Memorial Hospital, Mysore, India; 3 Medical Research Council Lifecourse Epidemiology Unit, Southampton General Hospital, Southampton, United Kingdom; Scientific Directorate, Bambino Hospital, Italy

## Abstract

**Objective:**

UK Indian adults have higher risks of coronary heart disease and type 2 diabetes than Indian and UK European adults. With growing evidence that these diseases originate in early life, we compared cardiometabolic risk markers in Indian, UK Indian and white European children.

**Methods:**

Comparisons were based on the Mysore Parthenon Birth Cohort Study (MPBCS), India and the Child Heart Health Study in England (CHASE), which studied 9–10 year-old children (538 Indian, 483 UK Indian, 1375 white European) using similar methods. Analyses adjusted for study differences in age and sex.

**Results:**

Compared with Mysore Indians, UK Indians had markedly higher BMI (% difference 21%, 95%CI 18 to 24%), skinfold thickness (% difference 34%, 95%CI 26 to 42%), LDL-cholesterol (mean difference 0.48, 95%CI 0.38 to 0.57 mmol/L), systolic BP (mean difference 10.3, 95% CI 8.9 to 11.8 mmHg) and fasting insulin (% difference 145%, 95%CI 124 to 168%). These differences (similar in both sexes and little affected by adiposity adjustment) were larger than those between UK Indians and white Europeans. Compared with white Europeans, UK Indians had higher skinfold thickness (% difference 6.0%, 95%CI 1.5 to 10.7%), fasting insulin (% difference 31%, 95%CI 22 to 40%), triglyceride (% difference 13%, 95%CI 8 to 18%) and LDL-cholesterol (mean difference 0.12 mmol/L, 95%CI 0.04 to 0.19 mmol/L).

**Conclusions:**

UK Indian children have an adverse cardiometabolic risk profile, especially compared to Indian children. These differences, not simply reflecting greater adiposity, emphasize the need for prevention strategies starting in childhood or earlier.

## Introduction

People of Indian origin migrating to the UK have experienced rates of coronary heart disease (CHD) and type 2 diabetes (T2D) which are markedly higher than those of the white European host population [1], [2] and those of their country of origin [3], though risks of CHD and T2D in India are now rising rapidly [4]. Although comparisons of cardiometabolic risk factors between Indian adult migrants and the white European host population have shown that adiposity and insulin resistance are higher among UK Indians [1], [5], assessment of the full extent of migration-related risk factor changes requires comparisons between Indians living in India and in the UK or other Western diaspora locations [6]. Such comparisons, few in number, have observed marked differences in LDL-cholesterol and blood pressure as well as adiposity and insulin resistance [6], [7]. In the most recent UK-based study, increased adiposity was identified as a key factor underlying migration-related changes in cardiometabolic risk [6].

CHD and T2D risks are influenced by factors operating in childhood, infant and fetal life [8], [9]. Earlier reports have suggested that differences in adiposity and insulin resistance between UK South Asians and UK white Europeans are apparent both in childhood [10]–[12] and adolescence [13], [14]. However, differences in cardiometabolic risk factors between Indian children living in India and the UK, and the contribution of adiposity, have been little studied [6]. We have therefore compared cardiometabolic risk factors among 9–10 year-old Indian children examined in comparable recent surveys in India and the UK; data on UK white European children have also been included for reference.

## Methods

Analyses were based on two studies, the Mysore Parthenon Birth Cohort Study (MPBCS) and the Child Heart and Health Study in England (CHASE). MPBCS is based on 663 normal births delivered at the CSI Holdsworth Memorial Hospital in Mysore, India during 1997–1998. 630 participants were eligible for follow up at approximately 9.5 years during 2007–2008. Ethical approval was obtained by the CSI Holdsworth Memorial Hospital ethical committee. Mysore is a burgeoning medium-scale city in southern India and a base to several traditional and home industries, and recently to a growing IT sector. The city has a population of one million which is mainly of middle socio-economic class. CHASE is a cross-sectional study of approximately 5000 9–10 year-old children who attended 200 Primary Schools in London, Birmingham and Leicester carried out between 2004 and 2007. The study population is multi-ethnic and included children of Indian and white European ethnic origins. Ethical approval was obtained from the Multicentre Research Ethics Committee. Full details of both studies are published elsewhere [10], [12], [15], [16]. In both studies, informed written consent was obtained from all parents or guardians and assent from participating children. Trained observers made standardized measurements of height, weight, waist and mid-upper arm circumference, triceps and subscapular skinfold thickness measurements using similar techniques. Arm-leg bioimpedance was recorded with a Bodystat Quadscan (MPBCS) or Bodystat 1500 (CHASE) (Bodystat Ltd, Isle of Man, UK) using the same validated equation in both studies to derive fat-free mass [17] and pubertal status assessed in girls using the Tanner breast development scoring system. Two seated measurements of blood pressure were made with a Dinamap 8100 (Critikon Inc, Tampa, Florida) in MPBCS or an Omron HEM-907 (Omron Healthcare, Inc., Kyoto, Japan) in CHASE. Appropriate cuff sizes were available in each study and room temperature was recorded. Dinamap 8100 blood pressures were calibrated to the standard mercury sphygmomanometer using pooled estimates derived from published calibration studies [18]–[20]; such adjustments were not needed for the Omron HEM-907 [21].

In both studies, blood samples were collected after overnight fasting. In MPBCS, EDTA plasma samples were frozen within 2–3 hours at −80°C and transferred to King Edward Memorial Hospital, Pune for analysis on study completion. Glucose, triglycerides, total and HDL cholesterol concentrations were analysed with standard enzymatic methods (Alcyon 3000 autoanalyzer; Abbott Laboratories) and plasma insulin with a time-resolved, fluoroimmunoassay (DELFIA) method (PerkinElmer Life and Analytical Sciences, Wallac Qy, Turku, Finland). In CHASE, plasma and serum samples were transferred to the Department of Clinical Biochemistry, Newcastle Hospitals NHS Trust within 48 h of collection. Plasma glucose was measured using the hexokinase method. Serum triglyceride, total and HDL-cholesterol were measured using an Olympus autoanalyser. Serum for insulin measurement was separated and frozen on dry ice after collection and analysed using an ELISA method which does not cross-react with proinsulin [22] in the Department of Medicine, University of Newcastle, UK. In both studies, LDL cholesterol was estimated using the Friedewald formula [23] and the homeostasis model assessment (HOMA) equations were used to provide estimates of insulin resistance and beta cell function [24]. To examine the influence of laboratory on blood marker patterns, EDTA samples from 26 CHASE participants stored at −70°C since collection were sent to the Pune laboratory for analysis following MPBCS protocols during 2010.

In CHASE, child ethnicity was defined using parental information on the self-reported ethnicity of both parents, or parental information on the ethnicity of the child. The ‘UK Indian’ group includes children whose parents both originated in India and ‘UK white European’ includes children whose ethnicity was defined as ‘white British,’ ‘white Irish,’ or ‘white European’

### Statistical methods

Statistical analyses were carried out using STATA/SE software (Stata/SE 10 for Windows, StataCorp LP, College Station, TX, USA). Variables were checked for normality and log transformed where necessary. Outcome variables which required log transformation included weight, BMI, waist and arm circumference, triceps and subscapular skinfolds, sum of skinfolds, triglyceride, fasting glucose and insulin, HOMA insulin resistance and beta cell function. The average age of the two study populations was slightly different and it was necessary to adjust for age as a confounder. The similarity of associations with age in the different study populations was formally examined; no marked differences in age slopes were observed and age adjustments were made using a single slope. Linear regression was used to create adjusted means and population differences, adjusting for age and sex (except in analyses where adjusted means are presented stratified by sex). Means were standardized to the average age. Adjusted geometric means and percentage differences were given for log transformed variables.

In order to examine whether the associations between BMI, fat mass percentage and sum of skinfolds differed between population groups, median spline plots were created using the MSPLINE command in Stata, which fits a smooth polynomial function to show the inter-relationships between BMI and fat mass percentage or sum of skinfolds (adjusted for age) in the different populations. Population differences in BMI at a given level of fat mass percentage (or sum of skinfolds) in different population groups were estimated using regression models which included an interaction term between population group and fat mass percentage or sum of skinfolds. Absolute differences in BMI were approximated from differences in log BMI by multiplying the proportional differences by the expected median BMI. These were estimated empirically by calculating the median BMI within 5 percentiles either side of the median for fat mass percentage or sum of skinfolds for all population groups combined.

Population differences in cardiometabolic risk markers were additionally adjusted for adiposity and height to examine whether these explained the differences observed, having first established that there were no consistent differences in the associations between adiposity, height and cardiometabolic risk markers between the three groups. Sensitivity analyses were conducted, in which girls with a Tanner breast development score greater than one were excluded from the analysis to remove girls who showed evidence of pubertal development. Paired mean or percentage differences and t-tests were used to quantify laboratory differences based on analyses of blood samples from the same individuals.

## Results

In MPBCS, 538 children (256 boys and 282 girls, mean age 9.4 years) participated and had full measurements (85% of all surviving birth cohort participants). In CHASE, 483 UK Indians and 1375 white Europeans (948 boys and 910 girls, mean age 10.0 years) participated (75.1% of Indians and 69.4% of white Europeans invited). Among the UK Indian children, most (83%) were born in the UK; 12% were born in India and 4% in other locations (for 1% birth place was unknown); their parental occupations were 31.3% managerial/professional, 33.8% intermediate and 26.5% in routine/manual; the remaining 8.4% were unemployed or had unknown occupations) [25]. Mean levels of adiposity and cardiometabolic risk factors are shown for boys and girls in each population group (Table 1) and overall population mean differences in these outcome measures (which were similar in boys and girls) in Table 2. Differences expressed as z-scores are presented in Supporting Information Table S1.

**Table 1:** Measures of body size/adiposity and cardiometabolic risk markers by population group and sex.

	Mean (95% CI)											
	UK Indian				UK white European				Indian			
	Boys (n = 248)		Girls (n = 235)		Boys (n = 700)		Girls (n = 675)		Boys (n = 256)		Girls (n = 282)	
Height (cm)	137.9	(137.1, 138.7)	138.5	(137.7, 139.4)	138.6	(138.1, 139.1)	138.1	(137.6, 138.7)	133.6	(132.7, 134.5)	133.2	(132.3, 134.1)
Trunk length (cm)	69.4	(68.9, 69.8)	69.7	(69.3, 70.2)	71.1	(70.8, 71.4)	71.0	(70.7, 71.3)	69.8	(69.3, 70.2)	69.5	(68.9, 70.0)
Leg length (cm)	68.6	(68.1, 69.0)	68.8	(68.3, 69.3)	67.5	(67.2, 67.8)	67.1	(66.8, 67.4)	63.8	(63.3, 64.4)	63.7	(63.2, 64.3)
Fat mass %	27.7	(26.6, 28.8)	30.5	(29.4, 31.6)	25.9	(25.3, 26.6)	28.8	(28.1, 29.4)	19.2	(18.0, 20.5)	24.2	(23.1, 25.4)
Systolic BP (mmHg)	103.7	(102.4, 104.9)	103.5	(102.2, 104.9)	104.8	(104.0, 105.6)	103.8	(103.0, 104.7)	94.2	(92.7, 95.6)	92.4	(91.0, 93.9)
Diastolic BP (mmHg)	63.0	(61.9, 64.1)	64.2	(63.0, 65.4)	61.8	(61.1, 62.5)	62.1	(61.4, 62.8)	56.6	(55.3, 57.8)	55.3	(54.1, 56.6)
HDL cholesterol (mmol/l)	1.56	(1.52, 1.60)	1.45	(1.41, 1.49)	1.55	(1.52, 1.57)	1.48	(1.45, 1.50)	1.12	(1.08, 1.16)	1.08	(1.04, 1.12)
LDL cholesterol (mmol/l)	2.81	(2.72, 2.90)	2.79	(2.69, 2.88)	2.66	(2.60, 2.72)	2.70	(2.65, 2.76)	2.29	(2.19, 2.38)	2.35	(2.26, 2.45)
Total cholesterol (mmol/l)	4.70	(4.60, 4.80)	4.64	(4.53, 4.75)	4.55	(4.48, 4.61)	4.58	(4.51, 4.64)	3.81	(3.70, 3.92)	3.88	(3.77, 4.00)

	Geometric mean (95% CI)											
Weight (kg)	33.3	(32.5, 34.3)	34.3	(33.4, 35.3)	34.9	(34.3, 35.5)	35.0	(34.4, 35.6)	26.2	(25.4, 27.0)	25.8	(25.1, 26.7)
BMI (kg/m^2^)	17.6	(17.2, 17.9)	17.9	(17.6, 18.3)	18.2	(18.0, 18.4)	18.4	(18.1, 18.6)	14.7	(14.4, 15.1)	14.6	(14.3, 15.0)
Waist circumference (cm)	62.2	(61.2, 63.2)	62.9	(61.8, 64.0)	63.9	(63.2, 64.5)	64.0	(63.3, 64.7)	55.2	(54.1, 56.2)	55.3	(54.3, 56.4)
Arm circumference (cm)	21.0	(20.6, 21.3)	21.5	(21.2, 21.9)	21.5	(21.3, 21.7)	22.0	(21.8, 22.3)	18.2	(17.8, 18.5)	18.3	(18.0, 18.6)
Triceps skinfold (mm)	12.1	(11.5, 12.7)	14.2	(13.6, 14.8)	11.9	(11.5, 12.2)	14.3	(13.9, 14.7)	8.7	(8.2, 9.2)	10.7	(10.2, 11.2)
Subscapular skinfold (mm)	9.2	(8.6, 9.8)	11.2	(10.5, 12.0)	8.1	(7.8, 8.4)	9.9	(9.5, 10.3)	7.0	(6.5, 7.5)	8.8	(8.2, 9.4)
Sum of skinfolds (mm)	21.5	(20.3, 22.7)	25.8	(24.5, 27.1)	20.2	(19.5, 20.9)	24.4	(23.6, 25.2)	15.8	(14.8, 16.8)	19.6	(18.5, 20.7)
Triglyceride (mmol/l)	0.8	(0.8, 0.9)	1.0	(0.9, 1.0)	0.7	(0.7, 0.8)	0.8	(0.8, 0.9)	0.8	(0.8, 0.9)	0.9	(0.9, 1.0)
Glucose (mmol/l)	4.6	(4.5, 4.6)	4.5	(4.4, 4.5)	4.5	(4.5, 4.6)	4.5	(4.4, 4.5)	4.7	(4.7, 4.8)	4.7	(4.6, 4.8)
Insulin (pmol/l)	49.4	(45.4, 53.7)	60.8	(55.8, 66.1)	39.2	(37.2, 41.3)	44.7	(42.5, 47.0)	18.6	(17.0, 20.4)	26.6	(24.5, 28.9)
Insulin resistance	0.9	(0.8, 1.0)	1.1	(1.0, 1.2)	0.7	(0.7, 0.8)	0.8	(0.8, 0.9)	0.3	(0.3, 0.4)	0.5	(0.5, 0.5)
Beta cell function	108.0	(102.4, 114.0)	129.3	(122.4, 136.5)	94.0	(90.9, 97.2)	106.5	(103.1, 110.1)	52.3	(49.4, 55.5)	67.6	(64.0, 71.3)

All means (geometric means for log transformed variables) were adjusted age and stratified by sex. Blood pressure was also adjusted for instrument and room temperature.

Missing values: n = 44 for fat mass %, n = 37 for blood pressure and n = 348 for all blood markers.

**Table 2:** Population differences in measures of body size/adiposity and cardiometabolic risk markers.

	Difference (95% CI)								
	UK Indian -			UK white European -			UK Indian -		
Outcome	Indian		p(diff)	Indian		p(diff)	UK white European		p(diff)
Height (cm)	4.8	(4.0, 5.7)	<0.0001	5.0	(4.2, 5.8)	<0.0001	−0.1	(−0.8, 0.5)	0.67
Trunk length (cm)	0.0	(−0.5, 0.4)	0.86	1.5	(1.0, 1.9)	<0.0001	−1.5	(−1.9, −1.1)	<0.0001
Leg length (cm)	4.9	(4.4, 5.4)	<0.0001	3.5	(3.1, 4.0)	<0.0001	1.4	(1.0, 1.8)	<0.0001
Fat mass %	7.3	(6.1, 8.5)	<0.0001	5.6	(4.5, 6.6)	<0.0001	1.7	(0.9, 2.6)	<0.001
Systolic BP (mmHg)	10.3	(8.9, 11.8)	<0.0001	11.0	(9.7, 12.3)	<0.0001	−0.7	(−1.8, 0.4)	0.20
Diastolic BP (mmHg)	7.7	(6.4, 8.9)	<0.0001	6.0	(4.9, 7.1)	<0.0001	1.7	(0.7, 2.6)	<0.001
HDL cholesterol (mmol/l)	0.41	(0.37, 0.45)	<0.0001	0.41	(0.38, 0.45)	<0.0001	−0.01	(−0.04, 0.03)	0.69
LDL cholesterol (mmol/l)	0.48	(0.38, 0.57)	<0.0001	0.36	(0.28, 0.45)	<0.0001	0.12	(0.04, 0.19)	0.002
Total cholesterol (mmol/l)	0.82	(0.71, 0.94)	<0.0001	0.71	(0.62, 0.81)	<0.0001	0.11	(0.02, 0.20)	0.01

	% Difference (95% CI)								
Weight (kg)	30.0	(26.2, 34.0)	<0.0001	34.3	(30.8, 37.9)	<0.0001	−3.2	(−5.3, −0.9)	0.01
BMI (kg/m^2^)	21.0	(18.2, 23.8)	<0.0001	24.6	(22.1, 27.1)	<0.0001	−2.9	(−4.6, −1.2)	<0.001
Waist circumference (cm)	13.2	(11.1, 15.3)	<0.0001	15.7	(13.8, 17.7)	<0.0001	−2.2	(−3.6, −0.8)	0.002
Arm circumference (cm)	16.5	(14.3, 18.7)	<0.0001	19.3	(17.4, 21.3)	<0.0001	−2.4	(−3.7, −1.0)	<0.001
Triceps skinfold (mm)	35.6	(28.8, 42.8)	<0.0001	34.9	(28.9, 41.2)	<0.0001	0.5	(−3.3, 4.5)	0.80
Subscapular skinfold (mm)	29.4	(20.6, 38.7)	<0.0001	13.9	(7.1, 21.2)	<0.0001	13.6	(7.7, 19.7)	<0.0001
Sum of skinfolds (mm)	33.7	(26.2, 41.7)	<0.0001	26.1	(19.9, 32.7)	<0.0001	6.0	(1.5, 10.7)	0.01
Triglyceride (mmol/l)	3.7	(−2.0, 9.8)	0.21	−8.3	(−12.7, −3.6)	<0.001	13.1	(8.2, 18.1)	<0.0001
Glucose (mmol/l)	−4.1	(−5.2, −2.9)	<0.0001	−4.8	(−5.7, −3.8)	<0.0001	0.8	(−0.1, 1.7)	0.10
Insulin (pmol/l)	145.2	(124.3, 168.0)	<0.0001	87.3	(73.3, 102.4)	<0.0001	30.9	(22.2, 40.2)	<0.0001
Insulin resistance	140.0	(119.5, 162.5)	<0.0001	84.3	(70.5, 99.3)	<0.0001	30.2	(21.5, 39.5)	<0.0001
Beta cell function	98.2	(87.2, 109.9)	<0.0001	67.8	(59.7, 76.4)	<0.0001	18.1	(13.0, 23.4)	<0.0001

All population differences (% percentage differences for log transformed variables) were adjusted for age and sex. Blood pressure was also adjusted for instrument and room temperature.

### Differences between Indian and UK Indian children

Compared with Mysore Indian children, UK Indians were taller (mainly reflecting greater leg length); heavier and more adipose (higher mean BMI, waist and arm circumferences, subscapular and triceps skinfolds and fat mass percentage). They had higher fasting insulin concentrations, insulin resistance, beta cell function, HDL-and LDL-cholesterol concentrations, and systolic and diastolic blood pressure. Their mean triglyceride levels were however similar to those of Mysore Indians, while glucose levels were lower. The largest percentage differences (∼145%) and z-score differences (1.3–1.5) were observed for fasting insulin, insulin resistance and beta-cell function. White Europeans generally showed similar, though slightly smaller, differences from Mysore Indians, except that their trunk length and leg lengths were greater and triglyceride levels were lower.

### Differences between UK Indian and UK white European children

Differences between UK Indians and white European children were less marked than those between UK Indians and Mysore Indians (Table 2). UK Indians had a similar mean height but were lighter and had lower mean BMI, waist and arm circumferences than white Europeans. However, UK Indians had higher mean fat mass % and skinfold thickness (particularly subscapular skinfold); fasting insulin, insulin resistance and beta-cell function were markedly higher, while triglyceride, diastolic (though not systolic) blood pressure, total and LDL-cholesterol were all slightly higher. Fasting glucose and HDL-cholesterol were similar. Again the largest differences, both percentage (∼30%) and z-score (∼0.4) were observed for fasting insulin, insulin resistance and beta cell function. The differences were similar in boys and girls and were little affected by adjustment for pubertal status in girls (data not presented). Differences between UK Indians and other population groups were similar in participants who were UK born and those who were not (data not presented).

### Population differences in the relationship between adiposity and BMI

**Figure 1:**
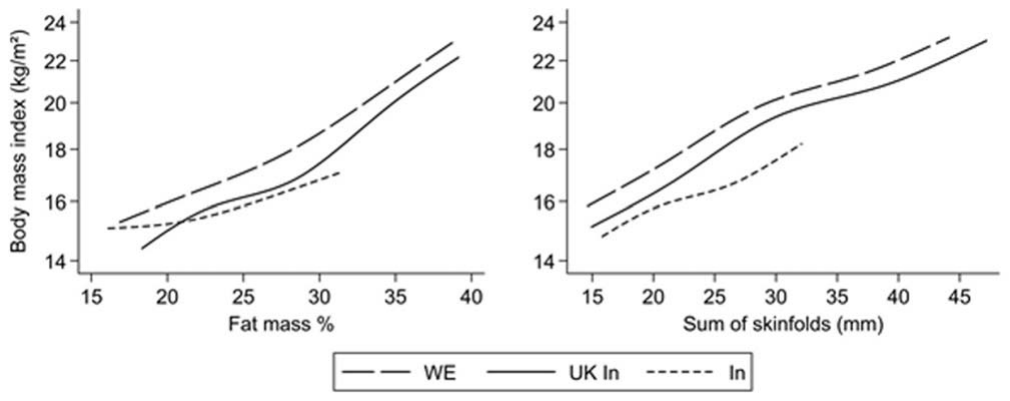
Interrelationships between measures of adiposity adjusted for age in white Europeans (long dashes), UK Indians (solid line) and Indians (short dashes) using median splines. Data are adjusted for age and presented between the 5^th^ and 95^th^ percentiles for the variable on the horizontal axis by removing the bottom 5^th^ and top 5^th^ centiles from each population group separately. The y-axis is on the log scale.

The inter-relations of BMI, fat mass % and sum of subscapular and triceps skinfolds in the three study populations are shown in Figure 1, with estimated absolute differences in BMI at the median fat mass percentage and sum of skinfolds shown in Table 3. For any given fat mass percentage (or sum of skinfolds), mean BMI was highest among white Europeans, intermediate among UK Indians and lowest among Mysore Indians, except at the lower end of the distribution where Mysore Indians had similar or higher BMI levels than UK Indians. Conversely, at any given BMI, fat mass percentage (or sum of skinfolds) was lowest among white Europeans, intermediate among UK Indians and highest among Indians. At the median fat mass percentage, UK white European children had a mean BMI ∼3.0 kg/m^2^ higher than Mysore Indian children, while UK Indians had a BMI level ∼1.8 kg/m^2^ higher than Mysore Indian children; these differences were larger in girls than boys. The corresponding BMI differences for sum of skinfolds were slightly smaller; again these differences were slightly larger in girls than boys (Table 3). In similar comparisons of UK Indians and white Europeans, UK Indians had a BMI level 1.0 kg/m^2^ lower than white Europeans both at the median fat mass percentage and the median sum of skinfolds; for fat mass percentage this difference was very slightly higher in boys than girls.

**Table 3:** Estimated population differences in BMI at median adiposity (fat mass % or sum of skinfolds) levels in whole study population: for all children and by sex.

Explanatory variable		Estimated absolute difference in BMI (95% CI)					
		UK Indian -		UK white European -		UK Indian -	
		Indian		Indian		UK white European	
Fat mass %	All	1.79	(1.51, 2.09)	2.96	(2.70, 3.24)	−1.00	(−1.17, −0.82)
	Boys	1.16	(0.77, 1.56)	2.38	(1.99, 2.77)	−1.07	(−1.28, −0.85)
	Girls	2.03	(1.59, 2.48)	3.14	(2.75, 3.54)	−0.94	(−1.23, −0.64)
Sum of skinfolds (mm)	All	1.50	(1.30, 1.71)	2.69	(2.50, 2.88)	−1.02	(−1.16, −0.88)
	Boys	1.30	(1.02, 1.58)	2.46	(2.20, 2.73)	−1.02	(−1.19, −0.84)
	Girls	1.65	(1.33, 1.97)	2.89	(2.61, 3.17)	−1.06	(−1.28, −0.83)

Estimated population differences were evaluated at the median level of adiposity (fat mass % or sum of skinfolds) and were adjusted for age and sex (except by sex), population group and an interaction term between population group and the adiposity marker. Population differences presented separately for boys and girls were from a stratified analysis by sex.

### Effect on population differences of adjusting for adiposity and height

Adiposity markers were strongly correlated with insulin, insulin resistance, triglyceride, LDL-cholesterol and blood pressure in all population groups (data not presented). The effects of adiposity adjustment (with fat mass percentage and sum of skinfolds) on the population differences in blood markers and blood pressure are shown in Table 4; percentage changes in the population differences in cardiometabolic risk markers after adjustment for adiposity are shown in Supporting Information Table S3. The differences between UK Indian and Mysore Indian children in mean levels of insulin, insulin resistance and beta cell function were reduced by at most one third, while differences in total and LDL-cholesterol and blood pressure were little affected; differences in glucose and HDL-cholesterol became more marked. All these differences remained highly statistically significant. Adjustment for adiposity had similar effects on UK Indian-white European differences, reducing those in insulin, insulin resistance and beta-cell function by up to one quarter, with smaller effects on differences in diastolic blood pressure, total and LDL-cholesterol and triglyceride. Again, all differences remained highly statistically significant.

**Table 4:** Population differences in cardiometabolic risk markers with and without adjustment for adiposity.

		Difference (95% CI)								
	Adjusted for adiposity? †	UK Indian -			UK white European -			UK Indian -		
Outcome		Indian		p(diff)	Indian		p(diff)	UK white European		p(diff)
Systolic BP (mmHg)	No	10.5	(9.1, 12.0)	<0.0001	11.1	(9.8, 12.4)	<0.0001	−0.5	(−1.6, 0.6)	0.35
	Yes	8.5	(7.1, 10.0)	<0.0001	9.6	(8.3, 10.9)	<0.0001	−1.0	(−2.1, 0.0)	0.06
Diastolic BP (mmHg)	No	7.8	(6.5, 9.0)	<0.0001	6.0	(4.9, 7.1)	<0.0001	1.8	(0.9, 2.7)	<0.001
	Yes	6.5	(5.2, 7.7)	<0.0001	5.0	(3.9, 6.1)	<0.0001	1.5	(0.5, 2.4)	0.002
HDL cholesterol (mmol/l)	No	0.41	(0.37, 0.45)	<0.0001	0.42	(0.38, 0.45)	<0.0001	−0.01	(−0.04, 0.02)	0.65
	Yes	0.46	(0.42, 0.50)	<0.0001	0.45	(0.42, 0.49)	<0.0001	0.01	(−0.03, 0.04)	0.74
LDL cholesterol (mmol/l)	No	0.48	(0.38, 0.58)	<0.0001	0.35	(0.27, 0.44)	<0.0001	0.12	(0.05, 0.20)	0.001
	Yes	0.41	(0.31, 0.51)	<0.0001	0.30	(0.22, 0.39)	<0.0001	0.11	(0.03, 0.18)	0.01
Total cholesterol (mmol/l)	No	0.82	(0.71, 0.94)	<0.0001	0.71	(0.61, 0.81)	<0.0001	0.12	(0.03, 0.21)	0.01
	Yes	0.78	(0.66, 0.89)	<0.0001	0.67	(0.57, 0.77)	<0.0001	0.10	(0.02, 0.19)	0.02

		% Difference (95% CI)								
Triglyceride (mmol/l)	No	4.3	(−1.5, 10.5)	0.15	−8.4	(−12.9, −3.8)	<0.001	13.9	(9.0, 19.1)	<0.0001
	Yes	−4.8	(−10.1, 0.7)	0.09	−14.6	(−18.6, −10.3)	<0.0001	11.4	(6.7, 16.2)	<0.0001
Glucose (mmol/l)	No	−4.2	(−5.4, −3.1)	<0.0001	−4.9	(−5.8, −3.9)	<0.0001	0.7	(−0.2, 1.6)	0.15
	Yes	−4.5	(−5.7, −3.3)	<0.0001	−5.1	(−6.1, −4.1)	<0.0001	0.6	(−0.3, 1.5)	0.20
Insulin (pmol/l)	No	146.8	(125.6, 170.0)	<0.0001	87.0	(73.0, 102.2)	<0.0001	32.0	(23.1, 41.5)	<0.0001
	Yes	103.1	(86.9, 120.7)	<0.0001	61.5	(50.3, 73.5)	<0.0001	25.8	(18.2, 33.9)	<0.0001
Insulin resistance	No	141.3	(120.5, 164.1)	<0.0001	84.0	(70.1, 99.0)	<0.0001	31.2	(22.3, 40.6)	<0.0001
	Yes	99.3	(83.3, 116.8)	<0.0001	59.3	(48.2, 71.2)	<0.0001	25.1	(17.5, 33.3)	<0.0001
Beta cell function	No	99.9	(88.7, 111.8)	<0.0001	68.1	(59.9, 76.6)	<0.0001	19.0	(13.8, 24.4)	<0.0001
	Yes	76.5	(67.3, 86.2)	<0.0001	53.0	(46.1, 60.2)	<0.0001	15.4	(10.8, 20.1)	<0.0001

All population differences (% percentage differences for log transformed variables) were adjusted for age and sex. Blood pressure was also adjusted for instrument and room temperature.

† Additional adjustment for adiposity (sum of triceps and subscapular skinfolds and fat mass %). N = 2318 for blood pressure and N = 2019 for all blood markers.

Height was strongly correlated with insulin, insulin resistance and blood pressure in all population groups. Adjustment for height in addition to adiposity (Supporting Information Table S2) led to slight further reduction in the differences between UK Indians and Mysore Indians in blood pressure, insulin, insulin resistance and beta-cell function, with no effects on total, LDL and HDL-cholesterol and an increase in the difference in fasting glucose; the differences however remained substantial and statistically significant. UK Indian-white Europeans differences were unchanged by additional adjustment for height.

### Laboratory comparison

Paired sample comparisons between the CHASE and MPBCS laboratories are summarized in Supporting Information Table S4. Mean insulin and triglyceride levels were slightly higher in the CHASE laboratory; the triglyceride difference was statistically significant. Total, LDL and HDL cholesterol levels were slightly higher in the MPBCS laboratory; the total cholesterol difference was statistically significant. The CHASE-MPBCS laboratory differences in insulin were substantially smaller than the observed study population differences and even the upper 95% confidence interval could not explain the observed population differences; laboratory differences in total, LDL and HDL cholesterol were small and opposite in direction to the observed population differences.

## Discussion

UK Indian children had substantially higher levels of adiposity, insulin resistance, LDL- and HDL-cholesterol and blood pressure than Mysore Indian children. They also had higher levels of adiposity, insulin resistance, LDL-cholesterol and diastolic blood pressure than UK white European children, though differences were less marked. At any given fat mass percentage, BMI was highest in white Europeans, intermediate in UK Indians and lowest in Mysore Indians. Conversely, at an equivalent BMI, body fat levels (based on fat mass percentage and skinfolds) were highest in Mysore Indians, intermediate in UK Indians and lowest among white Europeans. The differences in insulin concentrations and estimated insulin resistance and beta cell function between these population groups were modestly reduced by adjustment for adiposity markers (by approximately 30%); adjustment for adiposity had little impact on differences in other risk markers.

We are not aware of previous comparisons between Indian children in India and the UK, though previous adult studies have compared UK Punjabi migrants with siblings in the Punjab [7] and UK Gujaratis with Gujaratis in their villages of origin [6]. Those studies also showed higher levels of adiposity, insulin resistance, total-cholesterol and blood pressure in UK Indians. However, the differences in the present study appeared larger – fasting insulin concentration in UK Indian children was more than twice as high, compared with a one-third increase in the largest adult study [6], while differences in total and LDL-cholesterol (0.8 mmol/L, 0.5 mmol/L respectively) were also larger than previously reported in adults (0.5 mmol/L, 0.3 mmol/L respectively). Differences in systolic and diastolic blood pressure in the present study (10.3 mmHg, 7.7 mmHg respectively) were similar to those reported in adults (13.0 mmHg, 8.0 mmHg respectively) [6]. The higher HDL-cholesterol and the lower plasma glucose concentrations in UK Indians were also consistent with earlier findings in adults [6]. The differences between UK Indians and white Europeans (particularly in fat mass %, skinfold thickness and insulin resistance) are consistent with previous reports on UK South Asian children [10]–[12] and adults [5].

Strengths of the present investigation include the large size of the study populations (sufficient for the detection of modest risk factor differences) and the similarity of measurements and measurement techniques in CHASE and MPBCS, with scope for adjustment for method differences where present (e.g. for blood pressure). The equation used for deriving fat mass % from bioimpedance, though derived in white European children, has been validated in Indian children; it provided close agreement with estimates of fat mass % derived from doubly labeled water in girls, though it may have underestimated fat mass % in boys [26]. Both study laboratories were externally standardized; between-laboratory comparisons suggested that only a small component of the observed between-population differences in insulin and none of the observed differences in total, LDL and HDL cholesterol between Mysore and UK Indians could be explained by laboratory measurement differences. Adjustment for small differences in mean age between the study populations was undertaken. The observed differences between Indian and UK children are strongly coherent; adiposity differences expressed as SD scores are similar for a wide range of independently assessed markers, suggesting that the comparisons are valid. Although the two studies had different designs (CHASE was cross-sectional and MPBCS a birth cohort, both are effectively treated as cross-sectional studies in this investigation. The slight difference in the time periods in which these cross-sectional studies were carried out (CHASE between 2004 and 2007, MPBCS between 2007 and 2008, a median difference of 1 year 8 months) is unlikely to have an appreciable effect on the results. In supplementary analyses, we have modeled time trends in adiposity and cardiometabolic risk markers within the periods of the separate studies; no appreciable secular trends were apparent within each individual study. Both Indian and UK populations studied were urban, though both the Indian study (a birth cohort based on a hospital providing maternity care for the neighbouring population) and the UK study (a survey based on primary schools) are likely to have underrepresented children from extremely poor and extremely affluent families. Had the comparison been based on Indian children from semi-urban or rural settings, cardiometabolic differences between UK and Indian populations could have been even larger [27]. The regions of origin of the UK Indians (predominantly Gujarat and to a lesser extent Punjab, both in North India) are different from those of the Mysore population (based in the Karnataka region in Southern India). However, patterns of mean BMI and proportions of overweight or obesity in Indian adults in Karnataka are similar to those in Gujarat and those in India as a whole, though lower than those in Punjab [28]; rates of cardiovascular disease among Indian adults are similar in Karnataka and Gujarat regions but lower than those in Punjab [27]. However, the high levels of obesity and cardiovascular disease currently observed in Punjab are likely to have developed after the migration of families whose children participated in CHASE. Moreover, restriction of analyses in CHASE children to those specifically of Gujarat origin did not materially affect the findings of UK Indian-Indian comparisons.

The cardiometabolic risk comparisons between UK Indians and Mysore Indians complement those based on UK Indian-white European comparisons alone [1], [11]–[13]. If the markedly higher total (and LDL) cholesterol, blood pressure and adiposity levels in UK Indian children compared with Indians were to be maintained into adult life, these could account for approximately 22%, 44% and 29% higher CHD risks respectively at 40–49 years (potentially 2–3 fold combined) [29]–[31]. The higher BMI in UK Indians, if maintained into adulthood, could also account for a T2D risk approximately 60% higher than in Indians [30]. However, this could be an underestimate both because adiposity from childhood is likely to have a greater impact on T2D risk [32] and because the BMI difference between UK and Mysore Indians is likely to have underestimated the true difference in body fat (Figure 1).

Understanding the reasons for the population differences in adiposity and cardiometabolic risk is potentially important for prevention. Adiposity alone did not appear to explain the population group differences in cardiometabolic risk; this is consistent with the limited contribution of adiposity to differences in insulin resistance and blood lipids between UK South Asians and white Europeans in the main CHASE Study [12], [33]. The higher fat mass percentage at a given BMI observed among Indian children both in the UK and in Mysore is consistent with earlier observations in children [10] and adults [34] and with the concept of the ‘thin-fat’ Indian child [35]. The finding emphasizes the importance of using markers other than BMI for the assessment of adiposity in South Asian children [10]. Diet and physical activity are likely to play an important role in the population differences in adiposity and cardiometabolic risk observed. It is likely that differences in childhood diet (particularly higher intakes of calories and saturated fat) contribute to the higher levels of adiposity and circulating total, LDL and HDL-cholesterol and insulin levels in UK Indians [33]. However, it was not possible to examine this issue in detail in the present study because assessments of dietary intake were collected using very different methods in the two studies (24 hour recall in CHASE and food frequency questionnaire assessment in MPBCS). Low physical activity levels among UK Indians are also likely to contribute to higher levels of adiposity and insulin resistance [36]. Although we have shown in an earlier report that UK South Asians, including Indians, have lower objectively measured physical activity levels than UK white Europeans, it was not possible to examine this issue in detail in the present study because only 34% of the children in CHASE and 11% of children in MPBCS had objective physical activity assessments using an Actigraph GT1M. Among the children measured, mean levels of activity counts per minute (CPM) were lowest among UK Indians (mean 433.6, 95% CI 413.2, 454.0), intermediate among UK white Europeans (mean 492.5, 95% CI 483.3, 501.6) and highest among Mysore Indians (mean 515.8, 95% CI 476.5, 555.1). However, these differences, if representative of those in the populations studied, would not account for the pattern of cardiometabolic risk levels observed (markedly lower in Mysore Indians compared with both UK white Europeans and UK Indians), nor for their size, when taking account of the expected impact of these physical activity differences on cardiometabolic risk in the CHASE Study population [37]. This suggests that the contribution of physical activity to population differences in cardiometabolic risk may well be small. The contribution of other factors including socioeconomic status and family size to the risk marker differences between Mysore and UK Indians requires consideration, but is likely to be small. We have reported elsewhere that there are no consistent associations between socioeconomic status and cardiometabolic risk in UK Indians [25]. Although higher socioeconomic status is associated with higher levels of adiposity and insulin in Mysore Indians (data not presented), the population-wide differences between Mysore and UK Indians remain, even among participants with high socioeconomic status. Family size (measured in CHASE but not in MPBCS) is unrelated to cardiometabolic risk. Early life exposures, particularly low birth weight, are associated with T2D and insulin resistance [8], [38]. However, mean birth weights were lowest in Mysore Indian children [15], intermediate in UK Indians and highest in UK white Europeans, both in CHASE (Nightingale CM, unpublished data), suggesting that birth weight patterns alone do not account for the higher cardiometabolic risk of UK Indian children. Differences in family history of diabetes do not account for the population differences observed, which remained unchanged in a sensitivity analysis in which all children with parental or grand parental history of diabetes were excluded from analysis.

The early emergence of these adverse cardiometabolic risk profiles among UK Indian and white European children when compared with Mysore Indian children emphasizes that efforts to control chronic disease in the UK, especially in Indian diaspora populations need to take a life course approach, including children as well as adults. On this basis, population-wide improvements in diet (particularly to reduce total energy, saturated fat and salt intakes) and increases in physical activity levels are likely to be particularly important priorities in preventing the emergence of cardiometabolic risk in the next generation.

## Supporting Information

Table S1 Population differences in measures of body size/adiposity and cardiometabolic risk markers using z-scores.
(DOC)

Table S2 Population differences in cardiometabolic risk markers with and without adjustment for adiposity and height.
(DOC)

Table S3 Percentage change in population differences due to adjustment for adiposity.
(DOC)

Table S4 Laboratory differences in blood results for 26 subjects.
(DOC)
