# Supplementary material for: Cardiometabolic Risk Markers in Indian Children: Comparison with UK Indian and White European Children
Source: PLoS One. 2012 Apr 27;7(4):e36236. doi: 10.1371/journal.pone.0036236 (PMC3338673; doi:10.1371/journal.pone.0036236)
Supplement: Table S1 — Population differences in measures of body size/adiposity and cardiometabolic risk markers using z-scores. (DOC) [file pone.0036236.s001.doc]

Table S1: Population differences in measures of body size/adiposity and cardiometabolic risk markers using z-scores

|  | Difference in z-score | | | | | | | | |
| --- | --- | --- | --- | --- | --- | --- | --- | --- | --- |
|  | UK Indian - | |  | UK white European | |  | UK Indian - | |  |
| Outcome | Indian | | p(diff) | - Indian | | p(diff) | UK white European | | p(diff) |
| Height (cm) | 0.6 | (0.5, 0.8) | <0.0001 | 0.7 | (0.6, 0.8) | <0.0001 | 0.0 | (-0.1, 0.1) | 0.67 |
| Trunk length (cm) | 0.0 | (-0.1, 0.1) | 0.86 | 0.4 | (0.3, 0.5) | <0.0001 | -0.4 | (-0.5, -0.3) | <0.0001 |
| Leg length (cm) | 1.1 | (0.9, 1.2) | <0.0001 | 0.8 | (0.7, 0.9) | <0.0001 | 0.3 | (0.2, 0.4) | <0.0001 |
| Fat mass % | 0.8 | (0.7, 0.9) | <0.0001 | 0.6 | (0.5, 0.7) | <0.0001 | 0.2 | (0.1, 0.3) | <0.001 |
| Systolic BP (mmHg) | 0.9 | (0.8, 1.0) | <0.0001 | 0.9 | (0.8, 1.1) | <0.0001 | -0.1 | (-0.2, 0.0) | 0.20 |
| Diastolic BP (mmHg) | 0.8 | (0.7, 1.0) | <0.0001 | 0.6 | (0.5, 0.8) | <0.0001 | 0.2 | (0.1, 0.3) | <0.001 |
| HDL cholesterol (mmol/l) | 1.2 | (1.1, 1.3) | <0.0001 | 1.2 | (1.1, 1.3) | <0.0001 | 0.0 | (-0.1, 0.1) | 0.69 |
| LDL cholesterol (mmol/l) | 0.7 | (0.6, 0.9) | <0.0001 | 0.5 | (0.4, 0.7) | <0.0001 | 0.2 | (0.1, 0.3) | 0.002 |
| Total cholesterol (mmol/l) | 1.0 | (0.9, 1.1) | <0.0001 | 0.9 | (0.8, 1.0) | <0.0001 | 0.1 | (0.0, 0.2) | 0.014 |
| Weight (kg)* | 1.0 | (0.9, 1.1) | <0.0001 | 1.1 | (1.0, 1.2) | <0.0001 | -0.1 | (-0.2, 0.0) | 0.006 |
| BMI (kg/m^2^)* | 1.0 | (0.9, 1.1) | <0.0001 | 1.1 | (1.0, 1.3) | <0.0001 | -0.2 | (-0.2, -0.1) | <0.001 |
| Waist circumference (cm)* | 0.8 | (0.7, 0.9) | <0.0001 | 1.0 | (0.9, 1.1) | <0.0001 | -0.1 | (-0.2, -0.1) | 0.002 |
| Arm circumference (cm)* | 1.0 | (0.8, 1.1) | <0.0001 | 1.1 | (1.0, 1.2) | <0.0001 | -0.2 | (-0.2, -0.1) | <0.001 |
| Triceps skinfold (mm)* | 0.7 | (0.6, 0.9) | <0.0001 | 0.7 | (0.6, 0.8) | <0.0001 | 0.0 | (-0.1, 0.1) | 0.80 |
| Subscapular skinfold (mm)* | 0.5 | (0.4, 0.6) | <0.0001 | 0.2 | (0.1, 0.4) | <0.0001 | 0.2 | (0.1, 0.3) | <0.0001 |
| Sum of skinfolds (mm)* | 0.7 | (0.5, 0.8) | <0.0001 | 0.5 | (0.4, 0.6) | <0.0001 | 0.1 | (0.0, 0.2) | 0.01 |
| Triglyceride (mmol/l)* | 0.1 | (-0.1, 0.2) | 0.21 | -0.2 | (-0.3, -0.1) | <0.001 | 0.3 | (0.2, 0.4) | <0.0001 |
| Glucose (mmol/l)* | -0.5 | (-0.7, -0.4) | <0.0001 | -0.6 | (-0.7, -0.5) | <0.0001 | 0.1 | (0.0, 0.2) | 0.10 |
| Insulin (pmol/l)* | 1.3 | (1.2, 1.4) | <0.0001 | 0.9 | (0.8, 1.0) | <0.0001 | 0.4 | (0.3, 0.5) | <0.0001 |
| Insulin resistance* | 1.3 | (1.1, 1.4) | <0.0001 | 0.9 | (0.8, 1.0) | <0.0001 | 0.4 | (0.3, 0.5) | <0.0001 |
| Beta cell function* | 1.5 | (1.3, 1.6) | <0.0001 | 1.1 | (1.0, 1.2) | <0.0001 | 0.4 | (0.3, 0.5) | <0.0001 |

* Variable log transformed before z-scores calculated

All population differences (expressed as z-scores) were adjusted age and sex. Blood pressure was also adjusted for instrument and room temperature.
