# Supplementary material for: Cardiometabolic Risk Markers in Indian Children: Comparison with UK Indian and White European Children
Source: PLoS One. 2012 Apr 27;7(4):e36236. doi: 10.1371/journal.pone.0036236 (PMC3338673; doi:10.1371/journal.pone.0036236)
Supplement: Table S2 — Population differences in cardiometabolic risk markers with and without adjustment for adiposity and height. (DOC) [file pone.0036236.s002.doc]

Table S2: Population differences in cardiometabolic risk markers with and without adjustment for adiposity and height

|  | Adjusted for height and adiposity? † | Difference (95% CI) | | | | | | | | |
| --- | --- | --- | --- | --- | --- | --- | --- | --- | --- | --- |
|  |  | UK Indian - | |  | UK white European - | |  | UK Indian - | |  |
| Outcome |  | Indian | | p(diff) | Indian | | p(diff) | UK white European | | p(diff) |
| Systolic BP (mmHg) | No | 10.5 | (9.1, 12.0) | <0.0001 | 11.1 | (9.8, 12.4) | <0.0001 | -0.5 | (-1.6, 0.6) | 0.35 |
|  | Yes | 7.3 | (5.8, 8.7) | <0.0001 | 8.2 | (6.9, 9.5) | <0.0001 | -0.9 | (-2.0, 0.1) | 0.08 |
| Diastolic BP (mmHg) | No | 7.8 | (6.5, 9.0) | <0.0001 | 6.0 | (4.9, 7.1) | <0.0001 | 1.8 | (0.9, 2.7) | <0.001 |
|  | Yes | 6.1 | (4.8, 7.3) | <0.0001 | 4.6 | (3.5, 5.7) | <0.0001 | 1.5 | (0.6, 2.4) | 0.001 |
| HDL cholesterol (mmol/l) | No | 0.41 | (0.37, 0.45) | <0.0001 | 0.42 | (0.38, 0.45) | <0.0001 | -0.01 | (-0.04, 0.02) | 0.65 |
|  | Yes | 0.46 | (0.42, 0.51) | <0.0001 | 0.46 | (0.42, 0.50) | <0.0001 | 0.01 | (-0.03, 0.04) | 0.75 |
| LDL cholesterol (mmol/l) | No | 0.48 | (0.38, 0.58) | <0.0001 | 0.35 | (0.27, 0.44) | <0.0001 | 0.12 | (0.05, 0.20) | 0.001 |
|  | Yes | 0.44 | (0.34, 0.55) | <0.0001 | 0.34 | (0.25, 0.43) | <0.0001 | 0.10 | (0.03, 0.18) | 0.01 |
| Total cholesterol (mmol/l) | No | 0.82 | (0.71, 0.94) | <0.0001 | 0.71 | (0.61, 0.81) | <0.0001 | 0.12 | (0.03, 0.21) | 0.01 |
|  | Yes | 0.81 | (0.69, 0.93) | <0.0001 | 0.70 | (0.60, 0.81) | <0.0001 | 0.10 | (0.02, 0.19) | 0.02 |
|  |  | % Difference (95% CI) | | | | | | | | |
| Triglyceride (mmol/l) | No | 4.3 | (-1.5, 10.5) | 0.15 | -8.4 | (-12.9, -3.8) | <0.001 | 13.9 | (9.0, 19.1) | <0.0001 |
|  | Yes | -5.9 | (-11.2, -0.3) | 0.04 | -15.6 | (-19.7, -11.2) | <0.0001 | 11.4 | (6.8, 16.3) | <0.0001 |
| Glucose (mmol/l) | No | -4.2 | (-5.4, -3.1) | <0.0001 | -4.9 | (-5.8, -3.9) | <0.0001 | 0.7 | (-0.2, 1.6) | 0.15 |
|  | Yes | -4.8 | (-6.0, -3.6) | <0.0001 | -5.4 | (-6.4, -4.4) | <0.0001 | 0.6 | (-0.3, 1.5) | 0.18 |
| Insulin (pmol/l) | No | 146.8 | (125.6, 170.0) | <0.0001 | 87.0 | (73.0, 102.2) | <0.0001 | 32.0 | (23.1, 41.5) | <0.0001 |
|  | Yes | 91.3 | (75.9, 107.9) | <0.0001 | 51.6 | (41.0, 63.0) | <0.0001 | 26.2 | (18.6, 34.2) | <0.0001 |
| Insulin resistance | No | 141.3 | (120.5, 164.1) | <0.0001 | 84.0 | (70.1, 99.0) | <0.0001 | 31.2 | (22.3, 40.6) | <0.0001 |
|  | Yes | 87.8 | (72.7, 104.4) | <0.0001 | 49.7 | (39.1, 61.0) | <0.0001 | 25.5 | (17.9, 33.6) | <0.0001 |
| Beta cell function | No | 99.9 | (88.7, 111.8) | <0.0001 | 68.1 | (59.9, 76.6) | <0.0001 | 19.0 | (13.8, 24.4) | <0.0001 |
|  | Yes | 70.8 | (61.8, 80.2) | <0.0001 | 47.8 | (41.0, 54.8) | <0.0001 | 15.6 | (11.0, 20.3) | <0.0001 |

All population differences (% percentage differences for log transformed variables) were adjusted for age and sex. Blood pressure was also adjusted for instrument and room temperature.

† Additional adjustment for adiposity (sum of triceps and subscapular skinfolds and fat mass %) and height. N = 2318 for blood pressure and N = 2019 for all blood markers.
