# Supplementary material for: Cardiometabolic Risk Markers in Indian Children: Comparison with UK Indian and White European Children
Source: PLoS One. 2012 Apr 27;7(4):e36236. doi: 10.1371/journal.pone.0036236 (PMC3338673; doi:10.1371/journal.pone.0036236)
Supplement: Table S3 — Percentage change in population differences due to adjustment for adiposity. (DOC) [file pone.0036236.s003.doc]

Table S3: Percentage change in population differences due to adjustment for adiposity

|  | Percentage change difference due to adiposity adjustment | | |
| --- | --- | --- | --- |
|  | UK Indian - | UK white European - | UK Indian - |
| Outcome | Indian | Indian | UK white European |
| Systolic BP (mmHg) | -19.0 | -13.3 | 98.4 |
| Diastolic BP (mmHg) | -16.7 | -16.0 | -19.1 |
| HDL cholesterol (mmol/l) | 12.8 | 9.5 | -170.9 |
| LDL cholesterol (mmol/l) | -14.6 | -14.9 | -14.0 |
| Total cholesterol (mmol/l) | -5.9 | -5.2 | -10.3 |
| Triglyceride (mmol/l) | -212.4 | 72.3 | -18.3 |
| Glucose (mmol/l) | 6.3 | 4.1 | -10.3 |
| Insulin (pmol/l) | -29.8 | -29.4 | -19.4 |
| Insulin resistance | -29.7 | -29.4 | -19.4 |
| Beta cell function | -23.4 | -22.1 | -19.0 |
