# Supplementary material for: Cardiometabolic Risk Markers in Indian Children: Comparison with UK Indian and White European Children
Source: PLoS One. 2012 Apr 27;7(4):e36236. doi: 10.1371/journal.pone.0036236 (PMC3338673; doi:10.1371/journal.pone.0036236)
Supplement: Table S4 — Laboratory differences in blood results for 26 subjects. (DOC) [file pone.0036236.s004.doc]

Table S4: Laboratory differences in blood results for 26 subjects

|  | Laboratory mean | |  | |  |
| --- | --- | --- | --- | --- | --- |
|  | CHASE | MPBCS | Mean difference (95% CI) | | p(difference)† |
| HDL cholesterol (mmol/l) | 1.50 | 1.55 | -0.05 | (-0.10, 0.00) | 0.06 |
| LDL cholesterol (mmol/l) | 2.65 | 2.71 | -0.05 | (-0.12, 0.01) | 0.09 |
| Total cholesterol (mmol/l) | 4.50 | 4.57 | -0.07 | (-0.13, -0.00) | 0.04 |
|  | Laboratory geometric mean | |  |  |  |
|  | CHASE | MPBCS | % difference (95% CI) | | p(difference)† |
| Insulin (pmol/l)* | 53.2 | 50.0 | 6.4 | (-5.9, 20.4) | 0.31 |
| Triglycerides (mmol/l)* | 0.76 | 0.67 | 12.4 | (2.6, 23.1) | 0.01 |

* Variable log transformed

† P-values for differences are from t-tests
